# Targeting heme degradation pathway augments prostate cancer cell sensitivity to docetaxel-induced apoptosis and attenuates migration

**DOI:** 10.3389/fonc.2024.1431362

**Published:** 2024-07-18

**Authors:** Ramia J. Salloom, Iman M. Ahmad, Maher Y. Abdalla

**Affiliations:** ^1^ Department of Pathology, Microbiology, and Immunology, University of Nebraska Medical Center, Omaha, NE, United States; ^2^ Department of Clinical, Diagnostic, and Therapeutic Sciences, University of Nebraska Medical Center, Omaha, NE, United States

**Keywords:** heme oxygenase-1 (HO-1), prostate cancer (PC), SnPP (CID: 73755113), ZnPP, docetaxel, STAT 1, snail, vimentin

## Abstract

**Introduction:**

Chemotherapy, notably docetaxel (Doc), stands as the primary treatment for castration-resistant prostate cancer (CRPC). However, its efficacy is hindered by side effects and chemoresistance. Hypoxia in prostate cancer (PC) correlates with chemoresistance to Doc-induced apoptosis via Heme Oxygenase-1 (HO-1) modulation, a key enzyme in heme metabolism. This study investigated targeting heme degradation pathway via HO-1 inhibition to potentiate the therapeutic efficacy of Doc in PC.

**Methods:**

Utilizing diverse PC cell lines, we evaluated HO-1 inhibition alone and with Doc on viability, apoptosis, migration, and epithelial- to- mesenchymal transition (EMT) markers and elucidated the underlying mechanisms.

**Results:**

HO-1 inhibition significantly reduced PC cell viability under hypoxic and normoxic conditions, enhancing Doc-induced apoptosis through interconnected mechanisms, including elevated reactive oxygen species (ROS) levels, glutathione cycle disruption, and modulation of Signal Transducer and Activator of Transcription 1 (STAT1) pathway. The interplay between STAT1 and HO-1 suggests its reliance on HO-1 activation. Additionally, a decrease in cell migration and downregulation of EMT markers (vimentin and snail) were observed, indicating attenuation of mesenchymal phenotype.

**Discussion:**

In conclusion, the combination of HO-1 inhibition with Doc holds promise for improving therapeutic outcomes and advancing clinical management in PC.

## Graphical Abstract

In PC, both hypoxia and Doc induce the expression of HO-1, contributing to the blockade of ROS and resistance to therapy, ultimately promoting PC cell survival. The use of HO-1 inhibitors, such as ZnPP and SnPP, block HO-1 activity, elevates ROS levels, and sensitizes PC cells to the effects of Doc, leading to cellular apoptosis. Doc has been identified to induce the overexpression of STAT1 in PC cells, leading to chemotherapy resistance. Inhibiting STAT1 results in a suppression of HO-1 indicating that STAT1 works through HO-1 activation. So, inhibiting HO-1 enhances PC cellular sensitivity to Doc. Additionally, HO-1 inhibition diminishes the expression of Snail and vimentin, key components associated with EMT, resulting in reduced migratory capabilities of PC cells and a consequential attenuation of metastasis.

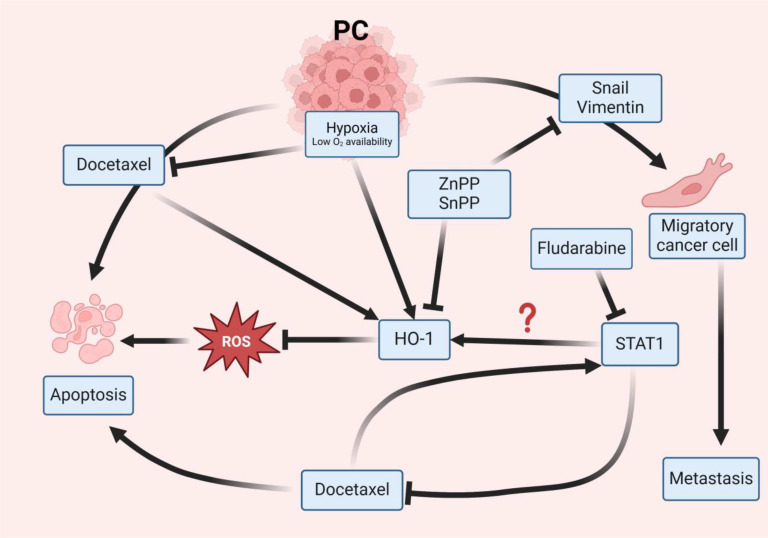

## 1 Introduction

PC is the most frequently diagnosed human carcinoma among men in western countries and ranks second in cancer-related mortality in the United States (1). Globally, it is the fourth most common cause of cancer fatality (2), with approximately 60% of PC cases detected predominantly in men aged over 65 years (3). While some PC progress slowly and remain localized within the prostate gland, causing minimal harm and responding to treatment through a combination of surgical and therapeutic interventions, others exhibit rapid growth and aggressiveness (3). The treatment landscape for PC is diverse and contingent upon the disease stage and grade (3). Androgen deprivation therapy (ADT) serves as the initial therapeutic recourse for PC and has shown efficacy as a primary treatment modality (4). However, despite its initial effectiveness, PC often transitions to a castration-resistant form (CRPC), characterized by heightened aggressiveness and resistance to ADT (5), eventually culminating in metastasis. This necessitates more effective therapeutic strategies, including the use of chemotherapeutic agents. Despite the availability of other approved therapies, it’s essential to recognize that PC is a heterogeneous disease, and treatment responses can vary widely among patients.

Docetaxel (Doc) is the most potent chemotherapeutic agent for CRPC (4). It is a powerful agent that binds to the β-tubulin subunit and locks the cell in the mitosis stage, causing a mitotic arrest at the G2/M phase of cell cycle, which leads to programmed cell death known as apoptosis (6, 7). Currently, Doc is highly efficacious for treating CRPC (8). However, Doc has many unfavorable side effects, and tumor cells develop resistance to the drug, which diminish its overall efficacy (4, 9). Innovative strategies may help achieve optimal therapeutic outcomes, minimize treatment-related toxicity, and improve patient quality of life. Chemoresistance to Doc is mediated by multiple molecular factors, including overexpression of heme oxygenase-1 (HO-1) in PC cells, which enhances cell proliferation and survival against oxidative stress caused by Doc (10).

Furthermore, the influence of hypoxia on Doc resistance in PC is a critical aspect of the treatment landscape (11). Hypoxia in PC is associated with a cascade of molecular events that not only enhances tumor’s aggressiveness but also contributes to chemotherapy resistance (12). One of the key players in this process is the upregulation of HO-1 (13). As mentioned earlier, HO-1 overexpression has been linked to PC chemoresistance. Under hypoxia, HO-1 induction is often intensified, resulting in an upsurge in heme degradation. This, in turn, leads to the enhanced production of anti-apoptotic molecules and free iron (Fe), which can aggravate oxidative stress (13, 14). The upregulation of HO-1 in response to hypoxia can contribute significantly to the development of resistance against the cytotoxic effects of Doc.

HO-1 is the inducible form of Heme Oxygenase. It is the enzyme that controls the first and the limiting step of heme degradation that is stimulated by oxidative stress to yield free iron (Fe), biliverdin, and carbon monoxide, which are anti-inflammatory products that promote cell survival (15). High levels of HO-1 have been shown to play a role in cancer development and resistance to therapy (15). In addition, our lab previously showed that HO-1 inhibition improves the responsiveness of pancreatic cancer cells to chemotherapy (16). Therefore, we hypothesized that combining Doc with HO-1 inhibitors holds the potential to improve PC treatment outcomes and reduce chemoresistance in PC cells.

Metalloporphyrins (MPs) are used as HO-1 inhibitors (17). Their structure is very similar to heme, consisting of protoporphyrin and a metal atom that replaces the iron in heme. MPs competitively bind to HO-1 with greater affinity than heme, effectively inhibiting HO-1 activity. Examples of Metalloporphyrin include zinc protoporphyrin (ZnPP) and tin protoporphyrin (SnPP). Both ZnPP and SnPP have been demonstrated to induce G1/S phase cell cycle arrest. However, this does not necessarily result in growth arrest, as cells may still proliferate at a slower rate (18). Conversely, cobalt protoporphyrin (CoPP) acts as an HO-1 inducer, stimulating HO-1 gene expression through the modulation of specific transcription factors (19).

In addition, the metastatic progression of PC involves dynamic cellular changes, particularly cell migration and EMT (20). EMT is a fundamental process in cancer metastasis, in which epithelial cells acquire mesenchymal characteristics, allowing them to migrate and invade the surrounding tissues (21, 22). These changes are often associated with increased aggressiveness and resistance to therapeutic interventions (23, 24). In PC, the acquisition of migratory capabilities by cancer cells is a critical determining factor for metastatic potential, further contributing to therapy resistance (25).

Studies have indicated a close interplay between Heme Oxygenase-1 (HO-1) expression and cellular behaviors associated with metastasis (26, 27). Elevated levels of HO-1 have been implicated not only in chemoresistance but also in facilitating the migratory and invasive properties of cancer cells (27, 28). HO-1-induced signaling pathways are known to intersect with those regulating cell migration and EMT, providing a mechanistic link between their upregulation and the metastatic phenotype (14, 15, 29).

By targeting the heme degradation pathway, our study demonstrated enhanced chemosensitivity in PC through HO-1 inhibition, suggesting a potential implication for therapy optimization.

## 2 Materials and methods

### 2.1 Gene expression analysis from Gene Expression Omnibus database

Gene expression analysis was conducted utilizing data from the Gene Expression Omnibus (GEO) (RRID: SCR_005012) database under accession numbers GSE30994 and GSE200879 GEO Accession viewer (nih.gov). These datasets were selected due to their focus on Homo sapiens PC tissue. GSE30994 dataset was classified into two groups: normal prostate tissues and tumor prostate tissues while GSE200879 dataset was classified into two distinct groups: normal prostate tissues and hormone-resistant prostate tumor tissues. We then extracted and compared the expression levels of HMOX-1 gene between these groups within each dataset. The normal prostate tissue group within each dataset was used as the control group in order to have base line expression references. The other groups were compared against the control groups to determine the relative change in gene expression.

### 2.2 Cell culture and reagents

PC cell lines, including LNCaP (RRID: CVCL_0395), DU145 (RRID: CVCL_0105), and C4–2B (RRID: CVCL_4784), were obtained from American Type Culture Collection and cultured in Roswell Park Memorial Institute (RPMI) 1640 medium (Fisher Scientific, Waltham, MA, USA, Cat# SH3002702) supplemented with 7% fetal bovine serum (Fisher Scientific, Waltham, MA, USA, Cat# MT35010CV), 1% penicillin streptomycin (Fisher Scientific, Waltham, MA, USA, Cat# 15140122), and 1% L-Glutamine (Fisher Scientific, Waltham, MA, USA, Cat #25030081). The cell cultures were maintained in a 5% CO2 incubator at 37°C. For experiments conducted under normoxic conditions, the cells were incubated in the HERACEL VIOS 160i humidified incubator (Fisher Scientific, Waltham, MA, USA) with a 5% CO2 at 37°C for 48 h. As for hypoxic exposure, the cells were incubated in a humidified hypoxia chamber at 2% O2 for 48 h. Other reagents used in this study were zinc protoporphyrin (ZnPP) (Santa Cruz Biotechnology, Dallas, TX, USA, Cat# sc-200329A), tin protoporphyrin IX dichloride (SnPP) (Santa Cruz Biotechnology, Dallas, TX, USA, Cat# sc-203452B), docetaxel (Fisher Scientific, Waltham, MA, USA, Cat#NC9968050), fludarabine (Fisher Scientific, Waltham, MA, USA, Cat# 349550), protoporphyrin IX cobalt chloride (CoPP) (Santa Cruz Biotechnology, Dallas, TX, USA, Cat# sc-294098), and hemin chloride (Hemin) (Santa Cruz Biotechnology, Dallas, TX, USA, Cat# sc-202646). All stock solutions of the reagents were dissolved in dimethyl sulfoxide (DMSO) for the *in vitro* study.

### 2.3 HO-1 knockdown cells

DU145 cells (RRID: CVCL_0105) and LNCaP cells (RRID: CVCL_0395) were transduced with lentivirus obtained from VectorBuilder Inc., Chicago, IL, USA, at a multiplicity of infection (MOI) ranging from 5 to 10. Transduction was performed according to the VectorBuilder protocol and enhanced using 1 μg/mL polybrene. Positively transduced PC cells were selected using RPMI medium supplemented with 4.0 μg/mL puromycin dihydrochloride hydrate in sterile water (Fisher Scientific, Waltham, MA, USA, Cat# AC227420100). Successful transfection was confirmed via western blot analysis using CoPP, a pharmacological HO-1 inducer, to compare its effect on KD with that of the parental cell lines.

### 2.4 HO-1 Tet-on cells

LNCaP cells (RRID: CVCL_0395) were co-transduced with two lentiviral vectors obtained from VectorBuilder (VectorBuilder Inc., Chicago, IL, USA): one containing TRE-driven gene of interest (GOI) expression and the other carrying the Tet regulatory protein expression vector. Transduction was enhanced using 1 μg/mL of polybrene. Lentiviral transduction followed the VectorBuilder protocol, and positively transduced cells were selected using RPMI media supplemented with 4.0 μg/mL puromycin dihydrochloride hydrate (Fisher Scientific, Waltham, MA, USA, Cat# AC227420100) and 1000 μg/mL hygromycin B in sterile water (Fisher Scientific, Waltham, MA, USA, Cat# AAJ6068103). To induce Tet-regulated gene expression, 0.50 μg/mL doxycycline (Dox) in sterile water (Fisher Scientific, Waltham, MA, USA, Cat# AAJ6042206) was employed. To confirm transfection, we conducted western blot analysis on Tet-on cell lysate with and without Dox and compared the HO-1 levels in these cells to those in the parental cells.

### 2.5 Western immunoblotting

Protein lysates were prepared using freshly prepared lysis buffer composed of 98% RIPA buffer (Fisher Scientific, Waltham, MA, USA, Cat# PI87787), 1% protease and phosphatase inhibitor (Fisher Scientific, Waltham, MA, USA, Cat# PI78441), and 1% EDTA. Protein quantification was performed using a DC Protein Assay Kit (Bio-Rad, Hercules, CA, USA, Cat# 5000111) following the manufacturer’s instructions and measured using a BioTek Synergy plate reader. Protein lysates (30 μg) were electrophoresed on a 12% Bis-Tris Gel (BioRad, Hercules, CA, USA, Cat# 4561044) at 100 V and transferred to Immobilon-P PVDF membranes (Fisher Scientific, Waltham, MA, USA, Cat# IPVH00010). The membranes were blocked using EveryBlot Blocking buffer (BioRad, Hercules, CA, USA, Cat# 12010020). Primary antibodies, including HO-1 pAb (Enzo Life Sciences, Farmingdale, NY, USA, Cat# BML-HC3001–0100, RRID: AB_11177779), β- Actin (13E5) rabbit mAb (Cell Signaling, MA, USA, Cat# 4970, RRID: AB_2223172), GAPDH (6C5) (Santa Cruz Biotechnology, Dallas, TX, USA, Cat# 32233, RRID: AB_627679), vimentin (D21H3) XP Rabbit mAb (Cell Signaling Technology, MA, USA, Cat# 5741, RRID: AB_10695459), and snail (Cell Signaling Technology, MA, USA, Cat# 3879, RRID: AB_2255011) were diluted 1:1000 in a 1:1 mixture of EveryBlot blocking buffer and 1% TBST and incubated overnight at 4°C. Secondary antibodies, goat anti-Rabbit IgG polyclonal secondary antibody (Enzo Life Sciences, Farmingdale, NY, USA, Cat# SAB-300J, RRID: AB_1505668) or Mouse IgG HRP-linked secondary antibody (Cell Signaling Technology, MA, USA, Cat# 7076S, RRID: AB_330924), were diluted 1:3000 in a 1:1 solution of EveryBlot blocking buffer and 1% TBST, and incubated for 1 h at room temperature with agitation. Blots were developed using Azure Biosystems Radiance Plus (VWR, Radnor, PA, USA, Cat# 10147–298) and Azure c600. β- Actin and GAPDH were utilized as housekeeping genes. Densitometry analysis of western blot bands was done using ImageJ software.

### 2.6 Quantitative real time polymerase chain reaction analysis

RNA extraction was conducted using the RNeasy mini kit (QIAGEN, Germantown, MD, USA, Cat# 74104) according to the manufacturer’s instructions. qPCR was performed using Power Up SYBR-Green Master Mix (Thermo Fisher Scientific, Waltham, MA, USA, Cat# A25776) and the plate was read using QuantStudio3. HMOX-1 mRNA levels were measured, and the relative mRNA levels were normalized to β-Actin using the ΔΔCt method. HMOX-1 mRNA primer pair used is F: CCAGGCAGAGAATGCTGAGTTC and R: AAGACTGGGCTCTCCTTGTTGC (OriGene, Rockville, MD, USA, Cat# HP205872), and the STAT1 primer pair used is F: ATGGCAGTCTGGCGGCTGAATT and R: CCAAACCAGGCTGGCACAATTG (OriGene, Rockville, MD, USA, Cat# HP210040).

### 2.7 Confocal microscopy

PC cells were plated in a 4-well slide chamber (BD Falcon, NJ, #354114) and treated with ZnPP, Doc, a combination of both, or Doc with N-acetylcysteine (NAC), an ROS scavenger (Thermo Fisher Scientific, Waltham, MA, USA, Cat# AC160280500). Fixation and permeabilization were performed using 4% paraformaldehyde (Fisher Scientific, Waltham, MA, USA, Cat# AAJ61899AK) and 0.1% Triton X-100 (Fisher Scientific, Waltham, MA, USA, Cat# MTX15681), respectively. Non-specific binding was blocked using 1% goat serum. The primary antibody against HO-1 (Enzo Life Sciences, Farmingdale, NY, USA, Cat# BML-HC3001–0100, RRID: AB_11177779) was diluted in 1% goat serum (BioRad, Hercules, CA, USA, Cat# C07SA) and incubated overnight at 4°C in a humidified chamber. The secondary antibody, Alexa Fluor 488 goat anti-rabbit IgG (H+L) (Thermo Fisher Scientific, Waltham, MA, USA, Cat# A-11034, RRID: AB_2576217), was employed. Vectashield DAPI mounting media (Vector Laboratories, San Fransisco, CA, USA, Cat# NC9029229) was used to mount the slides, and images were obtained using a Zeiss LSM 710 at the UNMC Advanced Microscopy Core Facility. Quantification analysis was done using ImageJ software.

### 2.8 Non-radioactive cell proliferation assay (MTT Assay)

The MTT cell proliferation assay was conducted using Promega’s MTT assay kit (Promega, Madison, WI, USA, Cat# G4000), according to the manufacturer’s instructions. Absorbance was measured after 48 h treatment at 570 nm using Tecan Spark 6.0.

### 2.9 Flow cytometry for apoptosis detection

PC cells treated with ZnPP, Doc, and their combination were assessed for apoptosis using the eBioscience Annexin V Apoptosis Detection kit (Invitrogen, Waltham, MA, USA, Cat# 509299) following the manufacturer’s instructions. Samples were processed at the UNMC Flow Cytometry Core Facility.

### 2.10 ROS detection

To detect ROS, PC cells were treated with ZnPP, Doc, or a combination of the two. Dichlorofluorescin diacetate (Thermo Fisher Scientific, Waltham, MA, USA, Cat# 28–781-0100MG) reagents were added to live cells, incubated at 37°C for 30 min, and imaged using a Zeiss LSM 800 at the UNMC Advanced Microscopy Core Facility. Quantification and image analysis was done using ImageJ software.

### 2.11 Glutathione assay (GSH)

Levels of oxidized glutathione (GSSG) and reduced glutathione (GSH) were assessed in PC cells treated with ZnPP, Doc, or a combination of ZnPP and Doc, as described in our earlier study (30). This assessment was performed using a GSH Quantification kit (DOJINDO Inc, Rockville, MD, USA, Cat# G257) according to the manufacturer’s guidelines.

### 2.12 Scratch assay

DU145 cells (RRID: CVCL_0105), LNCaP cells (RRID: CVCL_0395), and LNCaP KD cells were plated in 6-well culture plates at a density of 160,000 cells/well until they reached 80% confluency. A scratch was made using a sterile 200 μl pipette tip. Detached cells were washed with sterile phosphate-buffered saline (PBS), and fresh media was added to the wells. Representative images were captured at different time points (0, 24, and 48 h) and the gap width was quantified using EVOS FL Auto.

### 2.13 Statistical analysis

Data were analyzed using GraphPad Prism (RRID: SCR_002798) for windows (version 10.0). Statistical significance was considered for experiments with a P value of less than 0.05. Pairwise comparisons between groups were performed using 2-way ANOVA adjusted for three multiple comparisons with Tuki’s method and unpaired t-test. All data are representative of three independent experiments and are presented as the mean ± standard error of the mean.

## 3 Results

### 3.1 HMOX-1 gene expression analysis in PC development

**Figure 1:**
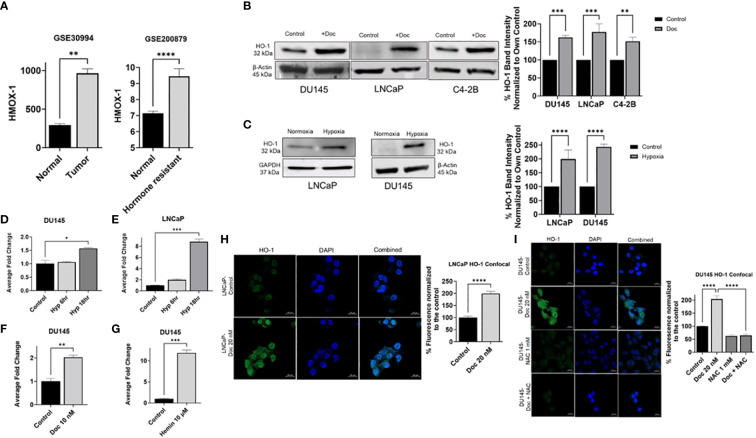
Hypoxia and chemotherapy induce HO-1 expression in different PC cell lines. A comparative analysis of HMOX-1 expression between normal prostate tissues group and tumor prostate tissues group using the dataset with accession number GSE30994, and between hormone resistant tissues group and the normal prostate tissues group using the dataset with the accession number GSE200879 from the GEO database **(A)**. Western blot and densitometry analysis showed that chemotherapy (Doc) induced HO-1 expression in DU145 (10 nM), LNCaP (10 nM), and C4–2B (0.5 μM) after 24 h treatment **(B)**. 24 h hypoxia (Hyp) induced HO-1 expression in LNCaP and DU145 cells **(C)**. qRT-PCR analysis showed that HMOX-1 gene expression was induced after 6 h and 18 h hypoxia but only significant at the 18 h exposure in DU145 and LNCaP cells **(D, E)**. qRT-PCR showed that 24 h treatment with Doc (10 nM) significantly induced HMOX-1 gene expression compared to non-treated control **(F)**. Hemin (10 μM) was used to validate the inducibility of HO-1 in our PC cell lines represented by DU145 **(G)**. Confocal analysis further confirmed our finding that 24 h treatment with Doc (20nM) induced HO-1 levels in LNCaP and DU145 cells and that using NAC (1 mM), an ROS scavenger, for 24 h treatment significantly reduced HO-1 levels in DU145 cells **(H, I)**. (n=3, **** = P<0.0001, *** = P<0.0001, ** = P<0.001, * = P<0.05).

To investigate the involvement of HO-1 in PC progression, we retrieved the PC-associated microarray datasets with the accession numbers GSE30994 and GSE200879 from the GEO database (31, 32). The first dataset with accession number GSE30994 was categorized into two groups: normal prostate tissues and tumor prostate tissues. Differential expression analysis indicated a significant upregulation of the HMOX-1 gene in tumor prostate tissues compared to normal prostate tissues (P < 0.05) (**Figure 1A**). Given that chemotherapy is often used as a next-line treatment when the cancer becomes resistance to hormone therapy, the dataset with accession number GSE200879 was partitioned into two distinct categories: normal prostate tissues and hormone-resistant prostate tissues. In this dataset, hormone-resistant prostate tissues exhibited significantly higher levels of HMOX-1 compared to normal prostate tissues (P < 0.05) (**Figure 1A**). This collective observation suggests a potential association between the increased expression levels of HMOX-1 and PC development.

By examining gene expression profile in normal, tumor, and hormone-resistance PC tissues, we can identify key molecular players, such as HMOX-1, that may contribute to PC development and therapy resistance. Understanding these mechanisms is essential for improving the efficacy of chemotherapy and developing targeted therapies to overcome resistance in advanced PC.

### 3.2 Hypoxia and Doc induces HO-1 expression in PC cells

To determine the role of HO-1 in PC, a comparative analysis of HO-1 levels in PC cells was conducted under various conditions. Specifically, HO-1 levels in PC cells under hypoxic conditions were compared to HO-1 levels in PC cells under normoxic conditions. HO-1 levels were also assessed after Doc treatment and compared with those in untreated control cells.

Various human PC cell lines were utilized in this study, including LNCaP cell line, which are derived from a lymph node metastasis of prostatic adenocarcinoma and are androgen sensitive. The C4–2b cell line, a bone-metastasis variant derived from LNCaP cells, metastasizes to bone, is androgen insensitive, and exhibits features of advanced PC progression, rendering it a valuable model for studying advanced disease stages. Additionally, DU145 cells line, derived from a brain metastasis of PC patient, is androgen-independent and is commonly used as a model for studying aggressive, hormone-refractory PC.

Western blot analysis revealed a significant overexpression of HO-1 at the protein level after Doc treatment compared to the control in DU145, LNCaP, and C42B cells (P < 0.05) (**Figure 1B**). Similarly, a significant increase in HO-1 levels was observed under hypoxia compared to normoxia in LNCaP and DU145 cell lines (P < 0.05) (**Figure 1C**).

Quantitative real-time PCR analysis (qRT-PCR) demonstrated similar results at the mRNA level, where HMOX-1 gene expression was significantly increased under hypoxia in both DU145 and LNCaP cells (P < 0.05) (**Figures 1D, E**). Furthermore, following Doc treatment, DU145 cells exhibited similar upregulation of HMOX-1 gene expression (P < 0.05) (**Figure 1F**). As a positive control, we employed Hemin, a recognized HO-1 inducer, which validated the inducibility of HO-1 in our cell lines (**Figure 1G**).

Confocal microscopy further confirmed our observations, revealing a significant increase in HO-1 levels following Doc treatment in both LNCaP and DU145 cells (P < 0.05) (**Figures 1H, I**). To confirm that HO-1 induction is mediated by ROS, N-acetyl cysteine (NAC), a synthetic ROS scavenger, was used (33). NAC significantly decreased the levels of HO-1 even after Doc treatment, strongly suggesting that Doc induces HO-1 expression in PC cells through an ROS-mediated mechanism (P < 0.05) (**Figure 1I**). Taken together, these results demonstrate that hypoxia and chemotherapy induce HO-1 expression in PC cells *in vitro*.

### 3.3 Inhibition of HO-1 activity leads to decreased PC cell viability and increased susceptibility to chemotherapy *in vitro*

**Figure 2:**
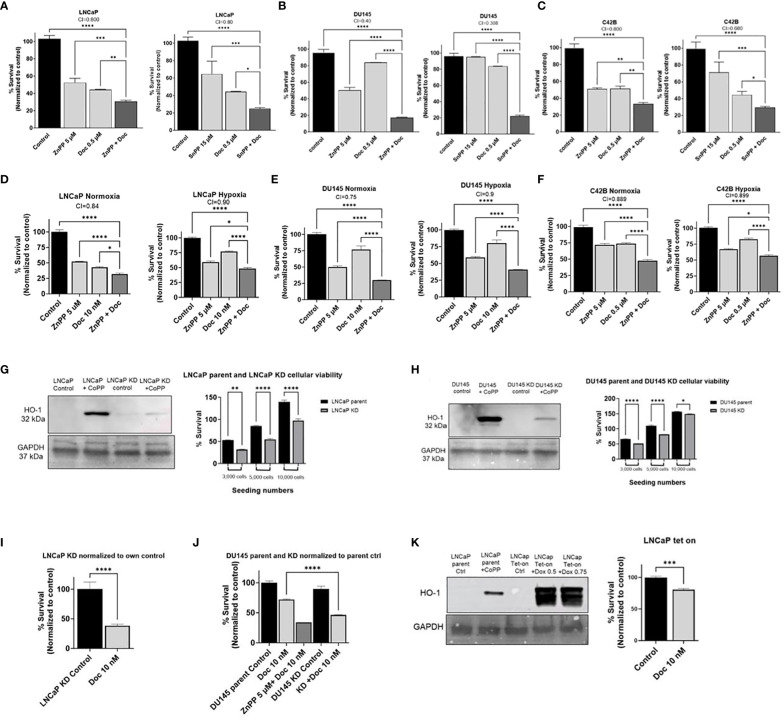
HO-1 inhibition decreases cellular viability and increases sensitivity to Doc in different PC cells. MTT analysis after 48 h treatment showed significant increase in PC cells sensitivity to Doc (0.5 μM) and significant decrease in cellular viability when using ZnPP (5 μM) and SnPP (15 μM) as our HO-1 inhibitors in LNCaP cells **(A)**, DU145 **(B)**, and C4–2B **(C)**. HO-1 inhibition had the same effect under 48 h hypoxic condition compared to normoxic conditions measured by MTT assay in LNCaP with 10 nM Doc and 5 μM ZnPP **(D)**, DU145 with 10 nM Doc and 5 μM ZnPP **(E)**, and C4–2B cells with 0.5 μM Doc and 5 μM ZnPP **(F)**. HO-1 KD, confirmed by western blot analysis employing CoPP (10 μM) as the HO-1 inducer, resulted in a significant decrease in cellular viability when seeded at different densities, 3000, 5000, and 10,000 cells, in comparison to the parental cells at the same seeding density measured by the MTT assay after 48 h in LNCaP cells **(G)** and in DU145 cells **(H)**. HO-1 KD increased PC cells sensitivity to Doc (10 nM) in LNCaP cells after 48 h treatment using MTT assay and normalized to own control **(I)**. DU145 HO-1 KD cells showed the same increase in sensitivity to Doc (10 nM) when compared to parental response and normalized to parental control after 48 h treatment **(J)**. LNCaP HO-1 Tet-On, confirmed by western blot analysis after 48 h treatment with Dox (0.5 μM and 0.75 μM) and compared to parental cells treated with CoPP (10 μM), further confirmed our findings and HO-1 induction resulted in an increase in PC cells resistance to the Doc (10 nM) treatment measured by the MTT assay after 48 h treatment represented in the bar graph **(K)**. Combination index (CI) values between Doc alone and Doc combined with ZnPP or SnPP are depicted showing synergistic effect (CI<1) or additive effect (CI=1). (n=3, **** = P<0.0001, *** = P<0.0001, ** = P<0.001, * = P<0.05).

Having established that hypoxia and Doc result in elevated HO-1 levels in PC cells, we next investigated whether inhibiting HO-1 will influence PC cellular viability and response to Doc. To study the synergistic/additive effects of Doc and HO-1 inhibitors, we treated PC cells with HO-1 inhibitors (ZnPP and SnPP), Doc, or a combination of both. Our results showed that the combination treatment resulted in a significant decrease in cellular viability and survival and increased PC cells sensitivity to Doc treatment compared to each treatment alone and to the control in LNCaP, DU145, and C4–2B cells (P < 0.05) (**Figures 2A–C**).

Next, we assessed the effect of HO-1 inhibition on PC cell viability and response to Doc under hypoxia compared to normoxia. Although hypoxia has been associated with increased resistance to Doc (13), we found that the combined therapy significantly reduced cellular viability and survival compared to each treatment alone and to the control measured by the MTT proliferation assay in LNCaP, DU145, and C4–2B cells (P < 0.05) (**Figures 2D–F**), respectively.

To further confirm that the inhibition of HO-1 is responsible for decreased PC cell viability and increased sensitivity to chemotherapy, we created LNCaP HO-1 knockdown (KD) cells, DU145 HO-1 KD cells, and LNCaP HO-1 Tet-on cells by lentiviral *in-vitro* transfection. We found that knocking down of the HO-1 gene significantly reduced cellular viability compared to parental cells at the same seeding density (P < 0.05) (**Figures 2G, H**). Despite the low or minimal expression of HO-1 protein under normoxic conditions, its inhibition disrupts critical protective mechanisms against oxidative stress and interferes with various cellular pathways, resulting in inhibited cell growth (34, 35). Moreover, treatment of KD cells with Doc increased the sensitivity of LNCaP and DU145 cells to our chemotherapy treatment (P < 0.05) (**Figures 2I, J**). On the other hand, inducing HO-1 in LNCaP cells using the Tet-on system significantly increased PC cell resistance to Doc (P < 0.05) (**Figure 2K**). Taken together, these data suggest that inhibition of HO-1 increases sensitivity to chemotherapy and reduces PC cell viability.

### 3.4 HO-1 inhibition and Doc combinational therapy increases PC cell sensitivity to Doc-induced apoptosis

**Figure 3:**
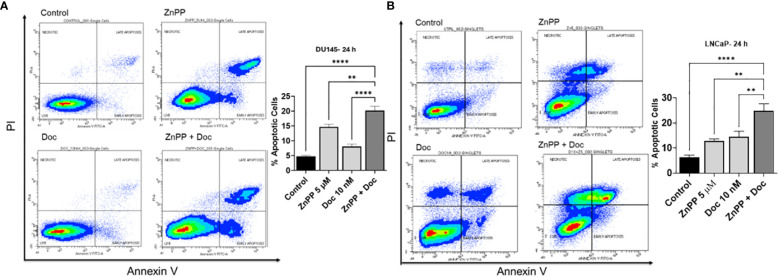
Combinational therapy of HO-1 and Doc enhances the response to apoptosis induced by Doc. Flow cytometry analysis revealed that the combined therapy of Doc (10 nM) and ZnPP (5 µM) significantly increased the number of apoptotic cells compared to each treatment separately and to the untreated control cells after 24 h treatment in DU145 **(A)** and LNCaP cells **(B)**. (n=3, **** = P<0.0001, ** = P<0.001).

Since Doc is a tubulin inhibitor that inhibits the growth of cancer cells and leads to apoptosis (6, 7), we then sought to investigate the effect of HO-1 inhibition on apoptosis. LNCaP and DU145 cells were treated with Doc and HO-1 inhibitor separately and in combination. Since both ZnPP and SnPP exhibited similar effects on the viability and response to Doc, we opted to use ZnPP as a representative HO-1 inhibitor in the following studies to maintain focus on our research objectives. The effect on apoptosis was evaluated using Annexin V/PI flow cytometry at the 24 h timepoint to capture the early cellular response to the combined treatment. We found that the combination therapy of HO-1 inhibitor, ZnPP, and Doc significantly increased the number of cells undergoing apoptosis compared to each treatment separately and compared to the untreated control cells in DU145 (**Figure 3A**) and LNCaP cells (**Figure 3B**). Based on these findings, HO-1 inhibition leads to a significantly higher proportion of PC cells undergoing apoptosis following exposure to Doc.

### 3.5 HO-1 inhibition increases the production of reactive oxygen species within PC cells and disrupts the glutathione cycle

**Figure 4:**
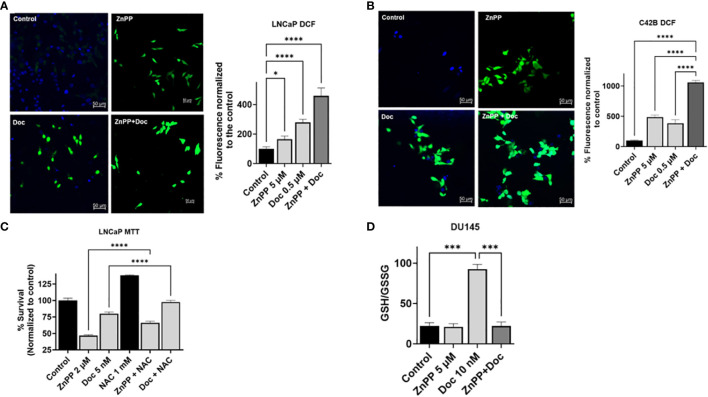
HO-1 inhibition increases ROS production in PC cells and disrupts the glutathione cycle. DCFDA assay demonstrated a significant increase in ROS levels with each treatment separately, ZnPP (5 µM) and Doc (0.5 µM) and was further induced with combinational treatment resulting in a significant increase in ROS levels compared to each treatment alone and to the control in LNCaP cells **(A)** and in C4-2B cells **(B)** following a 24 h treatment. For ROS quantification, ImageJ software was used. Employing NAC (1 mM), an ROS scavenger, significantly increased cell survival and reduced the efficacy of Doc (5 nM) treatment and ZnPP (2 μM) in LNCaP cells measured by MTT proliferation assay after 48 h treatment **(C)**. Combining HO-1 inhibitor, ZnPP (5 µM), with Doc (10 nM) resulted in disruption of glutathione homeostasis in DU145 cells following a 48 h treatment **(D)**. (n=3, **** = P<0.0001, *** = P<0.0001, * = P<0.05).

Since we found that HO-1 inhibition leads to a significant increase in apoptosis in PC cells, we investigated the cellular aspects affected by HO-1 inhibition that sensitize PC cells to Doc-induced apoptosis. Previously, our lab showed that HO-1 inhibition increased ROS production in pancreatic ductal adenocarcinoma (PDAC) (36). To this end, we studied the effect of HO-1 inhibition on ROS levels in PC cells using a Dichlorofluorescin Diacetate (DCFDA) assay to quantify ROS in live cells. ROS are highly reactive molecules that, when accumulated excessively, induce oxidative stress and cellular damage, leading to apoptosis (37). There were four groups: non-treated control, a group treated with ZnPP, a group treated with Doc, and a combined treatment group. We found that HO-1 inhibition significantly increased the production of ROS in LNCaP (P < 0.05) (**Figure 4A**) and C4–2B cells (P < 0.05) (**Figure 4B**). These elevated ROS levels render PC cells more responsive to the cytotoxic effects of Doc.

To confirm our finding that the combinational treatment with Doc and HO-1 inhibitors works through an ROS-mediated pathway, we used NAC. NAC is a synthetic antioxidant used as an ROS scavenger. The MTT assay results showed that using NAC and scavenging the ROS in LNCaP cells significantly increased PC cell viability and significantly reduced the effect of Doc treatment (P < 0.05) (**Figure 4C**).

To further analyze what could be a contributing factor to ROS accumulation in PC cells, we studied the effect of HO-1 inhibition on the glutathione cycle. Glutathione is a compound that exists in cells and is involved in antioxidation. It serves as an enzyme substrate for glutathione peroxidase and other enzymes. It is usually present in the reduced form (GSH) and is converted by oxidative stress via glutathione peroxidase to the oxidized form (GSSG) (38). The GSH/GSSG ratio is indicative of the oxidation level in the cells. To assess the effect on the glutathione cycle, we used a GSH assay kit and found that Doc treatment significantly converted the PC cell environment to a more reduced cellular environment in DU145 cells. This shift was attributed to the induction of HO-1 expression, serving as a form of cell adaptation mechanism associated with therapy resistance. However, HO-1 inhibition disrupted the glutathione cycle, resulting in the accumulation of ROS, and a significant reduction in the GSH/GSSG ratio in DU145 cells treated with the combination therapy compared to the group treated with Doc alone (P < 0.05) (**Figure 4D**). This disruption induced by HO-1 inhibition leads to the accumulation of ROS and the subsequent escalation of oxidative stress within PC cells, ultimately enhancing the responsiveness to Doc treatment.

### 3.6 HO-1 inhibition interferes with STAT1 signaling pathway and sensitizes PC cells to chemotherapy

Since we observed increased PC cell resistance to chemotherapy under hypoxia compared to normoxia, we decided to study the impact of HO-1 inhibition on signaling pathways that are involved in chemoresistance. STAT1 is a transcription factor that governs various cellular processes, including those related to apoptosis and inflammation. The STAT1 signaling pathway has been shown to play a role in chemoresistance in multiple cancers, including PC (39–41).

As chemotherapy and hypoxia increase the levels of STAT1 and HO-1 in PC cells, respectively, and STAT1 is involved in PC cell chemoresistance, we hypothesized that STAT1 is working through HO-1 activation.

**Figure 5:**
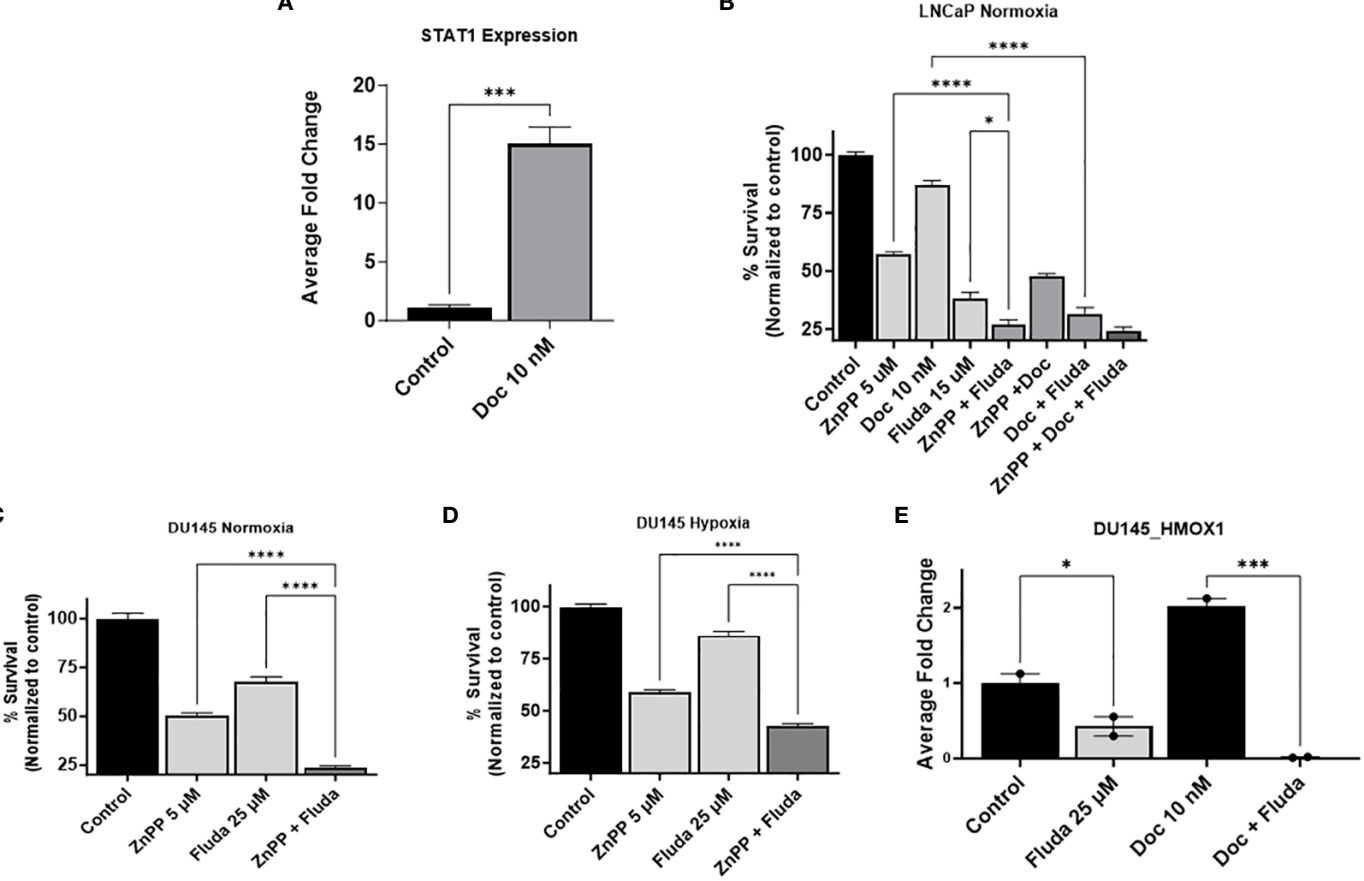
HO-1 inhibition disrupts the STAT1 signaling pathway. qRT-PCR analysis in LNCaP cells confirmed the higher expression level of STAT1 gene with the 24 h Doc (10 nM) treatment compared to untreated control cells **(A)**. MTT assay following a 48 h treatment showed that using Fluda (15 µM), a STAT1 specific inhibitor, significantly decreased PC cell survival when combined with either Doc (10 nM) or ZnPP (5 µM) in LNCaP cells under normoxia **(B)**, and in DU145 cells under both normoxic and hypoxic conditions **(C, D)**. qRT-PCR analysis revealed a significant decrease in HMOX-1 gene expression levels after 24 h treatment with Fluda (25 µM), alone or in combination with Doc (10 nM), in DU145 cells **(E)**. (n=3, **** = P<0.0001, *** = P<0.0001, * = P<0.05).

To study the interplay of HO-1 inhibition on the STAT1 pathway, we used fludarabine (Fluda), a specific STAT1 inhibitor that causes specific depletion of STAT1 protein, but not of the other STAT’s. qRT-PCR analysis confirmed the significantly heightened levels of STAT1 genes with Doc treatment compared to non-treated control cells in LNCaP cells (P < 0.05) (**Figure 5A**). The MTT assay results showed a significant decrease in cell survival when Fluda was combined with either the HO-1 inhibitor or Doc in LNCaP cells (P < 0.05) (**Figure 5B**). We also found that combining Fluda with HO-1 inhibitors in DU145 cells under hypoxic and normoxic conditions further decreased PC cell viability compared to each treatment alone (P < 0.05) (**Figures 5C, D**).

qRT-PCR analysis of the effect of STAT1 inhibitor on HMOX-1 gene expression further confirmed our hypothesis. Specifically, in DU145, using Fluda separately and in combination with Doc resulted in a significant decrease in HMOX-1 gene expression compared to the control group (P < 0.05) (**Figure 5E**).

These collective observations strongly suggest an interplay between STAT1 and HO-1, positioning STAT1 upstream of HO-1 and suggesting that it exerts its effects through the activation of HO-1. The specific impact on the STAT1 pathway implies that HO-1 inhibition plays an important role in modulating important signaling pathways, further enhancing the response of PC cells to Doc.

### 3.7 HO-1 inhibition reduces cell migration and attenuates the EMT capabilities in PC

**Figure 6:**
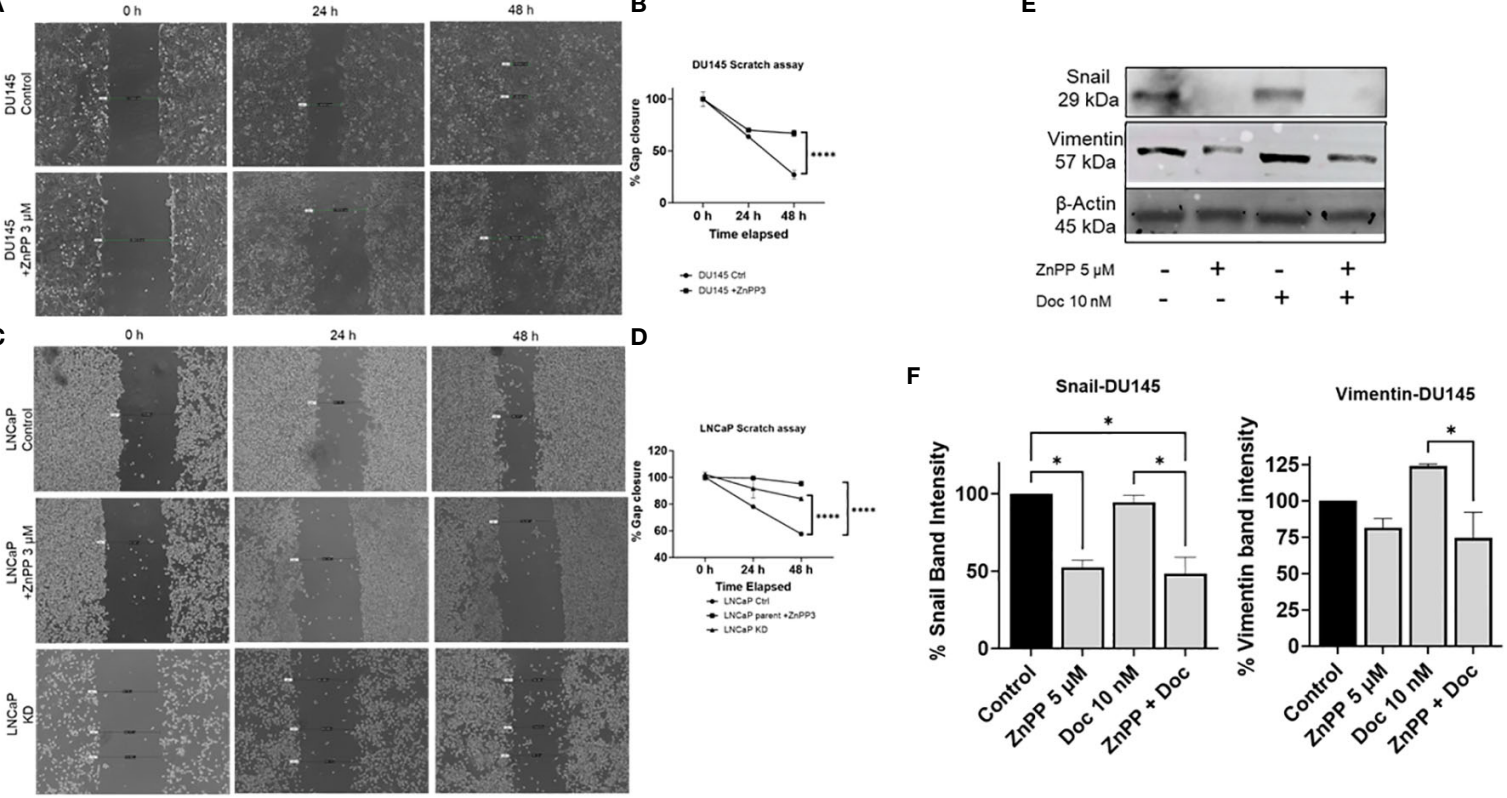
HO-1 HO-1 inhibition reduces PC cell migratory potential and affects the EMT capability in PC cells. Scratch assay showed that HO-1 inhibition and HO-1 KD cells significantly reduced PC cells migration. Treating DU145 cells with ZnPP (3 µM) and imaging the scratch at 0, 24, and 48 h post treatment significantly reduced PC cells migration, which correlates with percent gap closure, compared to the control untreated cells **(A, B)**. LNCaP cells treated with ZnPP (3 µM) and LNCaP HO-1 KD cells, both imaged at 0, 24, and 48 h, exhibited similar reduction in the rate of gap closure compared to non-treated control cells **(C, D)**. Representative Images of the control and treated groups at different time points, 0, 24, and 48 h, of the scratch assay **(A, C)**. The rate of wound closure at different time points **(B, D)**. Western blot and densitometry analysis showed decreased levels of EMT markers, Vimentin and Snail, with HO-1 inhibition in DU145 cells after 24 h treatment with Doc (10 nM) and ZnPP (5 µM) **(E, F)**. (n=3, **** = P<0.0001, * = P<0.05).

Metastasis is a critical factor in PC lethality. The transition of PC from a localized state to a metastatic state has a profound impact on patient’ outcomes and therapy response. Therefore, we aimed to study the effect of HO-1 inhibition on some aspects of metastatic. We evaluated the effect of HO-1 inhibition on PC cell migration, which directly correlates with the percentage of gap closure in the scratch assay. We found that HO-1 KD cells and cells treated with the HO-1 inhibitor (ZnPP) had a lower reduction rate in gap width compared to the parent cells. Quantitative analysis of these data revealed that treatment with HO-1 inhibitors significantly decreased DU145 and LNCaP cell migration relative to untreated control cells (*P* < 0.05) (**Figures 6A–D**). This decrease in cell migration was also evident in LNCaP HO-1 KD cells (**Figures 6C, D**). For this assay, we used ZnPP at a concentration lower than the IC50 that is normally used in other assays in order to determine the effect of HO-1 inhibition without killing the cells. While LNCaP cells predominantly display epithelial characteristics, the microenvironment surrounding the scratched area may offer cues conducive to cell migration, including signaling molecules and extracellular matrix components (42).

Next, we sought to explore the impact of HO-1 inhibition on key mesenchymal markers, snail and vimentin, which are both known to be highly expressed in DU145 cells (43–45). Using western blot analysis, we observed a significant reduction in snail protein expression in DU145 cells treated with the HO-1 inhibitor, ZnPP, compared to non-treated control cells (*P* < 0.05) (**Figures 6E, F**). Subsequently, when evaluating vimentin levels in DU145 cells, we noted a reduction in vimentin expression following treatment with the HO-1 inhibitor alone. Although this reduction did not reach statistical significance, it exhibited a consistent trend. Moreover, despite Doc treatment leading to an elevation in the expression of these markers, the combination of Doc with the HO-1 inhibitor significantly attenuated vimentin and snail levels in DU145 cells (*P* < 0.05) (**Figures 6E, F**).

Collectively, the data from these assays illustrate the efficacy of HO-1 inhibition in reducing the cellular migratory potential and attenuating the EMT capability in primary PC cells. This significantly impacts the metastatic progression of PC cells, presenting a promising avenue for therapeutic intervention.

## 4 Discussion

PC is characterized with a high fatality rate owing to resistance to therapies and poor prognosis (3). HO-1 plays a multifaceted role in PC and affects various aspects of disease’ progression. HO-1, an enzyme at the forefront of heme degradation, is often upregulated in response to oxidative stress, a common occurrence in cancer (46, 47). High levels of HO-1 have been implicated in PC progression where a significant upregulation of HO-1 was reported in HRPCA tissues relative to benign or localized prostate tissues (48). In addition, elevated HO-1 levels have been associated with increased PC cell proliferation, survival against oxidative stress, and resistance to chemotherapy, such as Doc (13). The capacity of this enzyme to generate free iron (Fe), along with anti-inflammatory products such as biliverdin and carbon monoxide, contributes to a microenvironment that promotes cancer cell survival and proliferation (13). However, the combination of HO-1 inhibitors with common chemotherapies for advanced PC has not been explored, and the effect of the hypoxic environment has not been considered in assessing treatment response. Thus, understanding and targeting heme degradation pathway through HO-1 inhibition represents a promising avenue to improve the management and treatment outcomes of PC.

Previously, our lab showed that HO-1 inhibition enhanced PDAC cell response to gemcitabine treatment (16). In this study, we explored the potential role of HO-1 in PC cell viability and sensitivity to Doc, under both hypoxic and normoxic conditions. Doc is one of the most effective cytotoxic agents that provides a significant advantage in the survival of patients with CRPC (49). It slows down the growth of cancer cells, leading to mitotic arrest and subsequent induction of apoptosis (50). However, side effects and chemoresistance render PC cells less responsive to Doc, and as a result, reduce their efficacy (51–53).

The primary outcome of our study highlights the pivotal role of HO-1 inhibition in reshaping the PC cell landscape. Using various PC cell lines *in vitro* to capitulate on the heterogeneity found in cancer cell populations, our study revealed that HO-1 inhibition using ZnPP and SnPP not only significantly reduced PC cell viability but also enhanced the overall response of PC cells to the cytotoxic effect of Doc under normoxic and hypoxic conditions. Additionally, our engineered HO-1 KD cell lines, LNCaP HO-1 KD and DU145 HO-1 KD, generated through *in vitro* lentiviral transfection, yielded similar findings, reinforcing the substantial impact of HO-1 knockdown on reducing PC cell viability and increasing sensitivity to chemotherapy. Conversely, the induction of HO-1 in our engineered LNCaP HO-1 Tet-on cells increased PC cell resistance to Doc, further confirming our findings.

Moreover, our data indicate that HO-1 inhibition modulates multiple cellular aspects, collectively augmenting the efficacy of Doc in PC. Our findings illustrate that HO-1 inhibition significantly amplifies the number of PC cells undergoing apoptosis. The observed increase in apoptotic events serves as a compelling indication that targeting and inhibiting HO-1 triggers multiple intracellular events culminating in apoptosis. Our study further revealed that HO-1 inhibition operates through an ROS-mediated pathway, as implicated by the significant increase in ROS levels following Doc treatment. The use of NAC, a synthetic ROS scavenger, increased cellular viability and reduced the response to Doc, indicating the pivotal role of ROS in this mechanism. Furthermore, HO-1 inhibitors disrupted the glutathione cycle resulting in a greater accumulation of ROS, rendering PC cells more susceptible to the effects of Doc.

Furthermore, our study revealed, for the first time, that HO-1 inhibition disrupts the STAT1 signaling pathway and demonstrates that STAT1 works through the activation of HO-1. This interplay between HO-1 and STAT1 represents a novel finding that further increases the sensitivity of PC cells to chemotherapy.

As PC advances, chemoresistance and metastasis play crucial roles in clinical outcomes. Therefore, investigating the impact of HO-1 inhibition on cell migration and EMT is an integral part of developing a more complete therapeutic approach. In our study, we demonstrated that HO-1 inhibition has a significant impact on the migratory potential and EMT capability of PC cells, as demonstrated by the reduced percentage rate of gap closure in the scratch assay and by the significant reduction in snail and vimentin expression when using the HO-1 inhibitor. These findings further demonstrate that combining HO-1 inhibitor with Doc has additional significance. HO-1 inhibition not only addresses chemoresistance, but also has the potential to impede metastasis by disturbing HO-1 mediated cellular behaviors associated with migration and invasiveness.

Our results demonstrate that by targeting HO-1 in PC cells and thereby disrupting the heme degradation pathway, we can diminish cellular survival, enhance the response to chemotherapy under both normoxic and hypoxic conditions, and mitigate certain aspects of metastasis. Thus, combining HO-1 inhibitors with chemotherapy provides a promising novel therapeutic approach that addresses the challenges of acquired resistance and metastasis in PC, ultimately improving chemotherapy outcomes for PC patients. This combination therapy deserves further *in vivo* and preclinical studies in relation to the PC response to chemotherapy to validate these observed effects and potentiate the exploration of this combination therapy in clinical trials.

## Data availability statement

The original contributions presented in the study are included in the article/supplementary material. Further inquiries can be directed to the corresponding author.

## Author contributions

RS: Conceptualization, Data curation, Formal Analysis, Investigation, Methodology, Project administration, Resources, Supervision, Validation, Visualization, Writing – original draft, Writing – review & editing. IA: Data curation, Methodology, Writing – review & editing. MA: Conceptualization, Funding acquisition, Methodology, Project administration, Supervision, Validation, Writing – review & editing.
